# Decoding the Lipid-Angiogram Link: Can Serum Lipid Profile Help Predict Your Clogged Arteries?

**DOI:** 10.7759/cureus.73454

**Published:** 2024-11-11

**Authors:** Siddharth Gupta, Akash Priyadarshi, Mujahid Beg, MU Rabbani

**Affiliations:** 1 Department of Medicine, Jawaharlal Nehru Medical College & Hospital, Aligarh Muslim University, Aligarh, IND; 2 Department of Cardiology, Jawaharlal Nehru Medical College & Hospital, Aligarh Muslim University, Aligarh, IND

**Keywords:** acs (acute coronary syndrome), cad: coronary artery disease, coronary angiography (cag), lipids, syntax score

## Abstract

Objective

In this study, we aimed to analyze the correlation between lipid profile and angiographic profile in terms of vessel involvement, segment involvement, and angiographic severity in patients with acute coronary syndrome (ACS).

Methods

One hundred patients diagnosed with ACS for the first time and undergoing coronary catheterization were included. Fasting samples for lipids profile were obtained. Data were analyzed to determine if lipid parameters were related to the severity of coronary artery disease (CAD), as measured by the SYNTAX score, and the number and location of affected vessels. The SYNTAX score offers a robust and reliable method for assessing the severity of CAD. By providing a comprehensive and quantitative evaluation, it empowers clinicians to make informed decisions about patient management and optimize treatment outcomes.

Results

Total cholesterol (TC), non-high-density lipoprotein (non-HDL) cholesterol, and apolipoprotein (Apo) B/A ratio correlated positively with the number of vessels involved, i.e., single, double, or triple vessel disease (SVD, DVD, and TVD). Apo B/A also showed a significant relationship with left anterior descending (LAD), left circumflex (LCX), and right coronary artery (RCA) involvement. RCA involvement had a significant association with TC and non-HDL levels. TC, non-HDL, and Apo B/A showed a significant relationship with mid-segment involvement. Apo A1 was found to have a significant inverse correlation, while Apo B, Apo B/A, and TC/HDL had a significant positive correlation with SYNTAX score class severity.

Conclusions

Our study revealed a strong association between lipid parameters and CAD severity, particularly in terms of vessel involvement and SYNTAX score. Elevated levels of TC, non-HDL cholesterol, and Apo B/A were linked to more extensive disease.

## Introduction

Coronary artery disease (CAD) is the leading cause of mortality worldwide. Estimates by the Global Burden of Disease study show a higher age-standardized cardiovascular (CVD) death rate of 272 per 100,000 population in India compared to the global average of 235, as per Prabhakaran et al. [1]. Clinical manifestation of CAD varies from stable angina, acute coronary syndrome (ACS), and heart failure to life-threatening sudden cardiac arrest. As per the INTERHEART-South Asia study by Ajay and Prabhakaran in 2010 [2], this rising burden of CAD is due to the increased prevalence of the following nine coronary risk factors: abnormal lipid profile, smoking, hypertension, diabetes, abdominal obesity, psychosocial factors, low fruits and vegetable consumption, and lack of physical activity. Pathologically, it occurs due to the mismatch between myocardial demand for blood and oxygen and its supply. The most common cause of this mismatch is atherosclerotic disease of epicardial coronary arteries, leading to plaque formation.

Studies in the past have predicted the risk and severity of CAD based on the lipid profile. The relationship between lipid profile and GENSINI score in type 2 diabetes mellitus patients was assessed by Du et al. [3], while Yu et al. compared different blood lipid parameters combined with carotid intima-media thickness (cIMT) in predicting CAD [4]. While the GENSINI score remains a useful tool for assessing the severity of CAD, the SYNTAX score is generally considered a more accurate and reliable predictor, especially in complex cases. It provides a more detailed assessment of the disease and can help clinicians make more informed decisions about patient management. On the other hand, the sensitivity and specificity of cIMT in the diagnosis of CAD is quite low. 

This study aims to explore the relationship between lipid profiles and angiographic indicators of CAD severity. It also strives to investigate the correlation among the lipid variables and the SYNTAX scoring method of CAD severity, as it may help us determine the possible future modality of treatment, i.e., percutaneous coronary intervention (PCI) vs. coronary artery bypass grafting (CABG), and may help in planning the management of CAD well ahead in time. Only a few studies in the literature have touched on these aspects so far, which makes our study relatively novel and unique.

## Materials and methods

Study design and data collection

This was a single-center, cross-sectional, hospital-based, observational study carried out from January 2020 to December 2021 comprising patients diagnosed and newly admitted with ACS for the first time. The screening for enrollment of patients in the study was conducted at the General Medicine and Cardiology OPD as well as the Emergency Department of the hospital. Data on lipid parameters of the patients were retrieved from the Central Lab, Department of Medicine at the hospital after analyzing their fasting blood samples. Coronary angiography reports were retrieved from the Cath Lab, Department of Cardiology.

Ethical approval

The study was approved by the Institutional Ethics Committee (Regd.), Jawaharlal Nehru Medical College & Hospital, Faculty of Medicine, Aligarh Muslim University (approval number: IECJNMC/586) as per the standards of Good Clinical Practice and the Helsinki Declaration (See Appendices). Written informed consent was obtained from all participants, who had the right to opt out of the study at any time.

Sample size

According to the study conducted by Deori et al. [5] in 2017-2018, the prevalence of CAD in Uttar Pradesh, India, was around 10%. At 95% CI and 5% margin of error, the sample size for this observational study came out to be 138.

Sample size(n) = $[(Z_{1-\alpha/2})^{2}(p)(q)]/(d)^{2}$

n = Desired sample size

Z_1−α/2 _= Critical value and a standard value for the corresponding level of confidence.

[At 95% CI or 5% level of significance (type-I error) it is 1.96 and at 99% CI it is 2.58]

P = Expected prevalence or based on previous research

q = 1-p

d = Margin of error or precision 

Participants

All patients presenting with ACS as per the fourth universal definition of myocardial infarction (MI) underwent screening for the study. Patients already diagnosed with CAD, diabetes mellitus, co-existing diseases such as inflammatory disease, sepsis, chronic kidney disease, malignancy, severe anemia, severe thrombocytopenia, lung pathologies along with those on lipid-lowering drugs, and those having any contraindications to coronary angiography procedure were excluded. Only those presenting for the first time with ACS who did not fit the aforementioned exclusion criteria were enrolled in the study.

**Figure 1:**
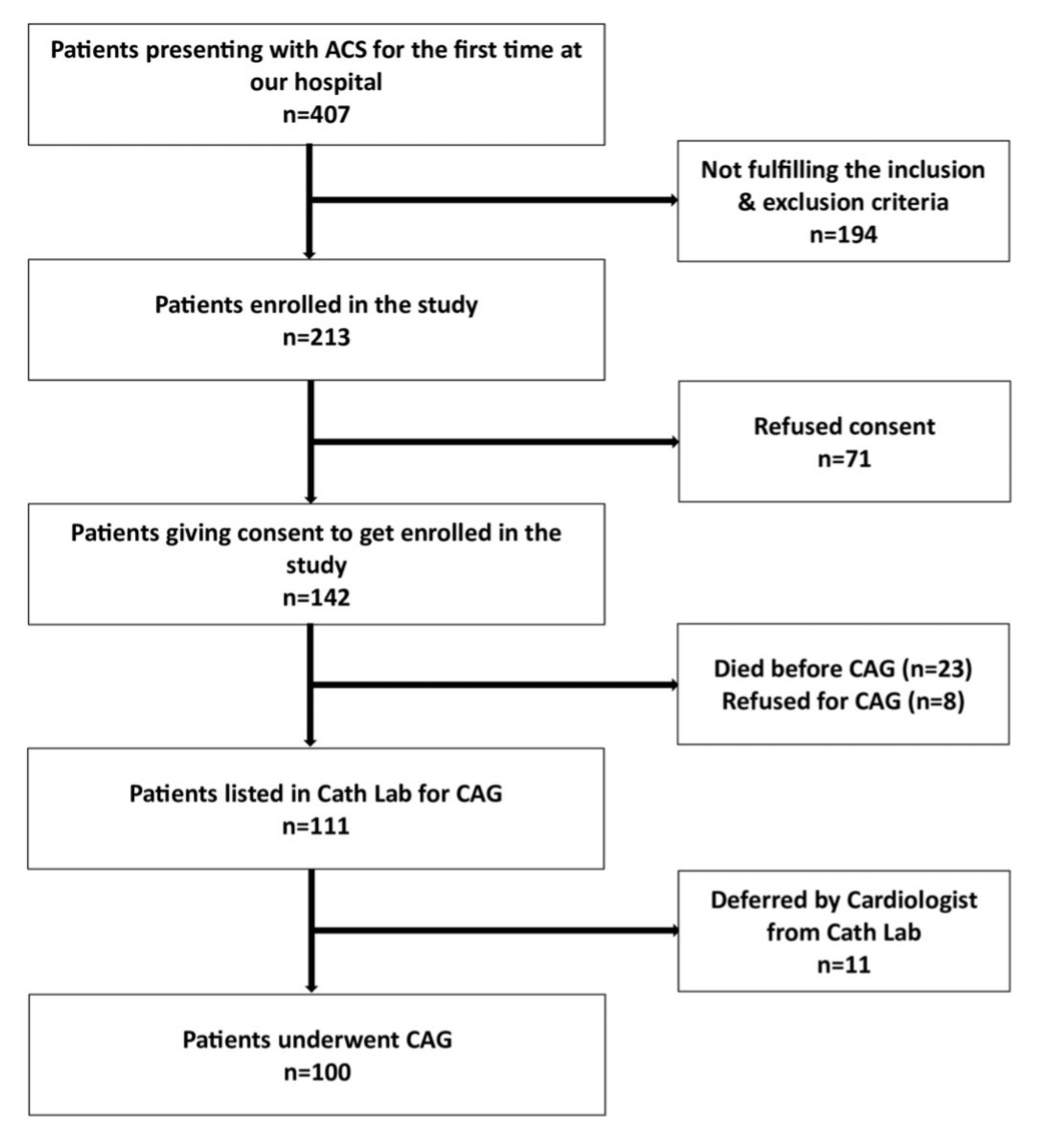
Flowchart depicting the selection of study participants ACS: acute coronary syndrome; CAG: coronary angiogram

As depicted in Figure 1, of the 407 potential participants, 213 were found eligible. However, due to various reasons, such as refusal of consent, death, financial constraints, severe illnesses, and the coronavirus disease 2019 (COVID-19) pandemic, only 100 patients were ultimately enrolled in the study.

The selection was done using strict inclusion and exclusion criteria and the participants were selected by one examiner. Fasting (>8 hrs.) blood samples for serum lipids were taken and patients underwent coronary catheterization. 

Instruments

Total cholesterol (TC) measurement was based on the principle of the cholesterol esterase oxidase-peroxidase method. The intensity of the red color developed, when read at 520 nm, was directly proportional to the TC concentration. Triglyceride (TG) levels were measured using a standard laboratory method that involved a color-based reaction. The intensity of the red color produced was directly related to the amount of triglycerides in the blood sample when read at 540 nm. High-density lipoprotein (HDL) levels were estimated using the principle of precipitation by buffer polyethylene glycol. HDL fraction remains in the supernatant which is then analyzed by an enzymatic assay method. The assay procedures were performed according to the directives in the kit manufactured by Ranbaxy Diagnostic Division, New Delhi, India in 2021. Low-density lipoprotein (LDL) was then calculated using the Friedewald-Levy-Fredrickson formula: LDL (mg/dl) = [TC-(TG/5)+HDL].

Apolipoprotein A-1 (Apo A1) and apolipoprotein B (Apo B) assessments were done based on the principle of turbidimetry following the directives in the kit manufactured by Euro Diagnostic Division of Peerless Biotech Private Limited, New Delhi, India in 2021. Lipoprotein (a) [Lp(a)] was estimated using the principle of Latex turbidimetry. The assay procedures were per the directives in the kit (Euro Diagnostic Division of Peerless Biotech Private Limited, New Delhi). All these kits were run on BioLis 24i® analyzer. 

All study patients underwent Coronary catheterization either by trans-femoral or trans-radial routes in the Cath Lab, Department of Cardiology, JNMCH, AMU. A coronary angiogram (CAG) was performed using Philips Cath Lab. CAG reports were prepared and cross-examined by four expert cardiologists. The severity of CAD was assessed using the SYNTAX-I score. SYNTAX-I score was calculated online on the official website: http://syntaxscore.org/calculator. Patients were then subcategorized into low (≤16), intermediate (16-22), and high (≥22) SYNTAX score classes. 

The reports were prepared by a qualified professional with expertise in the domain. The reports were cross-checked via duplicate testing and blinding of samples along with adherence to quality control charts. In case of any discrepancy, a consensus was reached through discussion and by referring to verifiable data sources. The reports and methods used were calibrated as per established benchmarks. The researcher who prepared the report had an experience of more than two decades in their relevant field.

Study outcome

The primary endpoint of the study was to analyze the correlation between lipid profile variables and angiographic parameters such as significant atherosclerosis, single vessel disease (SVD) vs. double vessel disease (DVD) vs. triple vessel disease (TVD), individual vessel involvement, segment involvement, and SYNTAX-I score.

Statistical analysis

All the qualitative variables such as vessel and segment involved were analyzed using the Pearson Chi-Square test, as it is a powerful tool for analyzing categorical data and understanding the relationship between different variables. On the contrary, all the quantitative variables like levels of various lipid parameters and SYNTAX score classes among different groups were analyzed using an independent sample t-test and Kruskal Wallis one-way ANOVA test. Along with this Pearson product-moment, correlation analysis was also used to assess the correlation among different variables. All statistical tests were performed using SPSS Statistics version 25.0 (IBM Corp., Armonk, NY). A p-value <0.05 was considered statistically significant.

## Results

Demographic and clinical characteristics

Among this study of 100 patients: 80 (80%) were males and only 20 (20%) were females, with a mean age of 53.24 ± 10.83 years. Females were older (57.35 ± 8.18 years) than their male counterparts (52.21 ± 11.23 years), as shown in Table 1. Individuals aged more than 45 years were prevalent in both sexes. ST-segment elevation myocardial infarction (STEMI) was the most common diagnosis (n = 65, 65%), while unstable angina (UA) was the least (n = 7, 7%) as depicted in Table 2. Smoking and hypertension were found in 47 (47%) and 34 (34%) of the study patients (Table 1). 

**Table 1:** Demographic characteristics of the study population SD: standard deviation

Parameter	Male	Female	Total
Participants, n (%)	80 (80%)	20 (20%)	100 (100%)
Age, years, mean ± SD	52.21 ± 11.23	57.35 ± 8.18	53.24 ± 10.83
Young (≤45 years), n (%)	30 (93.8%)	2 (6.2%)	32 (100%)
Old (>45 years), n (%)	50 (73.5%)	18 (26.5%)	68 (100%)
Smokers, n (%)	44 (93.6%)	3 (6.3%)	47 (100%)
Non-smokers, n (%)	36 (67.9%)	17 (32.1%)	53 (100%)
Hypertension, n (%)	26 (76.5%)	8 (23.5%)	34 (100%)
Dyslipidemia, n (%)	80 (80%)	20 (20%)	100 (100%)

**Table 2:** CAD and angiographic parameters in the study population CAD: coronary artery disease; double vessel disease; LAD: left anterior descending artery; LCX: left circumflex artery; LM: left main; NSTEMI: non-ST-elevation myocardial infarction; RCA: right coronary artery; STEMI: ST-segment elevation myocardial infarction; SVD: single vessel disease; TVD: triple vessel disease; UA: unstable angina

Parameter		N (%)	Chi-squared value	P-value
UA		7 (7%)	51.74	<0.001
NSTEMI		28 (28%)		
STEMI		65 (65%)		
Significant atherosclerosis		89 (89%)	57.69	<0.001
Right dominance		85 (85%)	120.14	<0.001
Left dominance		7 (7%)		
Co-dominance		8 (8%)		
SVD		52 (52%)	24.8	<0.001
DVD	LAD + LCX	6 (6%)		
	LAD + RCA	13 (13%)		
	LCX + RCA	2 (2%)		
TVD		17 (17%)		
Vessel involved	LM	7 (7%)	73.96	<0.001
	LAD	68 (68%)	12.96	< 0.001
	LCX	31 (31%)	14.44	< 0.001
	RCA	46 (46%)	0.64	> 0.05
Segment involved	Ostium	18 (18%)	40.96	< 0.001
	Proximal	64 (64%)	7.84	< 0.01
	Mid	46 (46%)	0.64	> 0.05
	Distal	28 (28%)	19.36	< 0.001
SYNTAX class	Low	79 (79%)	95.66	< 0.001
	Intermediate	16 (16%)		
	High	5 (5%)		

The mean values of TC, TG, LDL, HDL, non-HDL, Apo A1, Apo B, Lp(a), Apo B/A, and TC/HDL are shown in Table 3. Twelve (12%) patients had higher TC while 56 (56%) had elevated TG levels. LDL was elevated in 66(66%) patients, non-HDL in 47 (47%) patients, whereas 69 (69%) patients had lower HDL. Of note, 68 (68%) patients had raised Lp(a) levels while Apo B was raised in only 43 (43%) patients when compared to optimal values.

**Table 3:** Mean values of various lipid parameters in the study population Apo A1: apolipoprotein A1; Apo B/A: apolipoprotein B/A; HDL: high-density lipoprotein; LDL: low-density lipoprotein; Lp(a): lipoprotein (a); TC: total cholesterol; TG: triglycerides; SD: standard deviation

Parameters	Mean (mg/dL)	SD
TC	163.58	37.54
TG	169.51	67.84
LDL	116.30	40.03
HDL	37.12	10.65
Non-HDL	126.46	35.38
Apo A1	92.50	24.88
Apo B	79.56	21.93
Lp(a)	43.45	22.73
Apo B/A	0.91	0.34
TC/HDL	4.69	1.52

Significant atherosclerosis was present in 89 (89%) subjects; right coronary dominance was the most common type (n = 85, 85%). SVD was the most common finding (n = 52, 52%) followed by DVD (n = 21, 21%); only 17 (17%) were found to have TVD. In DVD, the LAD artery with RCA was the most common combination involved (Table 2).

**Figure 2:**
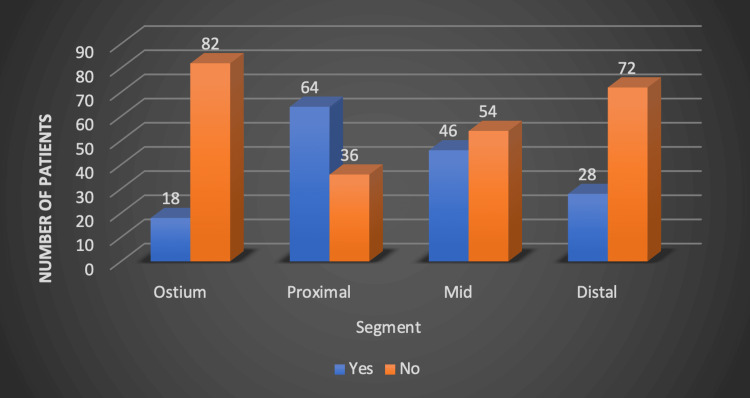
Distributions of segments involved

Among the 100 study subjects, a total of 152 vessels were significantly involved, with LAD being the most commonly involved vessel in 68 (68%) patients followed by RCA in 46 (46%) patients and LCX in 31 (31%) patients; LM was the least commonly involved (n = 7, 7%) patients. Among these 152 vessels, 194 segments had significant involvement. Of these, 81 (41.7%) were proximal segments, 62 (32%) were mid-segments, and 32 (16.5%) were distal; ostial involvement was seen in only 19 (9.8%). Proximal segment involvement was the most common (n = 64, 64%) followed by mid (n = 46, 46%). On the other hand, distal segment involvement was observed in only 28 (28%) patients, as shown in Figure 2. Seventy-four (74%) patients required stenting either in single or multiple vessels.

Lipid-angiogram correlation

Vessel Involvement

TC, non-HDL, and Apo B/A showed a statistically significant association with significant atherosclerosis (p<0.01). TC (r-value = 0.25, p<0.05), non-HDL (r-value = 0.26, p<0.05), and Apo B/A (r-value = 0.52, p<0.01) were found to have a significant positive correlation with the number of vessels involved (SVD, DVD, and TVD). Apo B/A demonstrated significant association with LAD, LCX, and RCA involved. TC, non-HDL, and Apo B/A showed significant association with RCA involvement as depicted in Table 4. 

**Table 4:** Serum lipid profile parameters in significant RCA involvement Apo A1: apolipoprotein A1; Apo B/A: apolipoprotein B/A; HDL: high-density lipoprotein; LDL: low-density lipoprotein; Lp(a): lipoprotein (a); RCA: right coronary artery; TC: total cholesterol; TG: triglycerides

Lipid profile	RCA involvement	Mean	Std. deviation	t-value	P-value
TC	Yes	174.26	36.06	2.709	<0.01
	No	154.48	36.66		
TG	Yes	162.98	71.75	-0.888	>0.05
	No	175.07	64.48		
LDL	Yes	120.98	31.82	1.08	>0.05
	No	112.31	45.81		
HDL	Yes	36.67	9.36	-0.385	>0.05
	No	37.50	11.71		
Non-HDL	Yes	137.59	35.03	3.02	<0.01
	No	116.98	33.12		
Lp(a)	Yes	45.80	21.90	0.957	>0.05
	No	41.44	23.42		
Apo B/A	Yes	1.01	0.37	2.751	<0.01
	No	0.83	0.29		
TC/HDL	Yes	4.99	1.55	1.828	>0.05
	No	4.44	1.46		

Segment Involvement

TC, non-HDL, and Apo B/A showed a significant association with mid-segment involvement as shown in Table 5. TC and non-HDL showed a significant association with distal segment involvement (Table 6).

**Table 5:** Serum lipid profile parameters in mid-segment involvement Apo A1: apolipoprotein A1; Apo B/A: apolipoprotein B/A; HDL: high-density lipoprotein; LDL: low-density lipoprotein; Lp(a): lipoprotein (a); TC: total cholesterol; TG: triglycerides

Lipid profile	Mid-segment involvement	Mean (mg/dL)	Std. deviation	t-value	P-value
TC	Yes	171.7	42.86	2.027	<0.05
	No	156.67	31.08		
TG	Yes	170.96	74.06	0.196	>0.05
	No	168.28	62.74		
LDL	Yes	116.37	34.57	0.016	>0.05
	No	116.24	44.48		
HDL	Yes	37.26	10.44	0.122	>0.05
	No	37	10.91		
Non-HDL	Yes	134.43	41.84	2.117	<0.05
	No	119.67	27.38		
Lipo-a	Yes	47.89	21.49	1.826	>0.05
	No	39.66	23.26		
Apo B/A	Yes	1.02	0.38	3.064	<0.01
	No	0.82	0.27		
TC/HDL	Yes	4.9	1.75	1.301	>0.05
	No	4.51	1.28		

**Table 6:** Serum lipid profile parameters in distal segment involvement Apo A1: apolipoprotein A1; Apo B/A: apolipoprotein B/A; HDL: high-density lipoprotein; LDL: low-density lipoprotein; Lp(a): lipoprotein (a); TC: total cholesterol; TG: triglycerides

Lipid profile	Distal segment involvement	Mean (mg/dL)	Std. deviation	t-value	P-value
TC	Yes	177.5	30.39	2.366	<0.05
	No	158.17	38.82		
TG	Yes	164.5	74.64	-0.459	>0.05
	No	171.46	65.46		
LDL	Yes	124.39	27.85	1.265	>0.05
	No	113.15	43.63		
HDL	Yes	38.21	9.17	0.639	>0.05
	No	36.69	11.2		
Non-HDL	Yes	139.29	28.95	2.31	<0.05
	No	121.47	36.56		
Lipo-a	Yes	47.55	20.14	1.127	>0.05
	No	41.85	23.59		
Apo B/A	Yes	1	0.31	1.566	>0.05
	No	0.88	0.35		
TC/HDL	Yes	4.82	1.14	0.53	>0.05
	No	4.64	1.65		

SYNTAX score correlation

**Figure 3:**
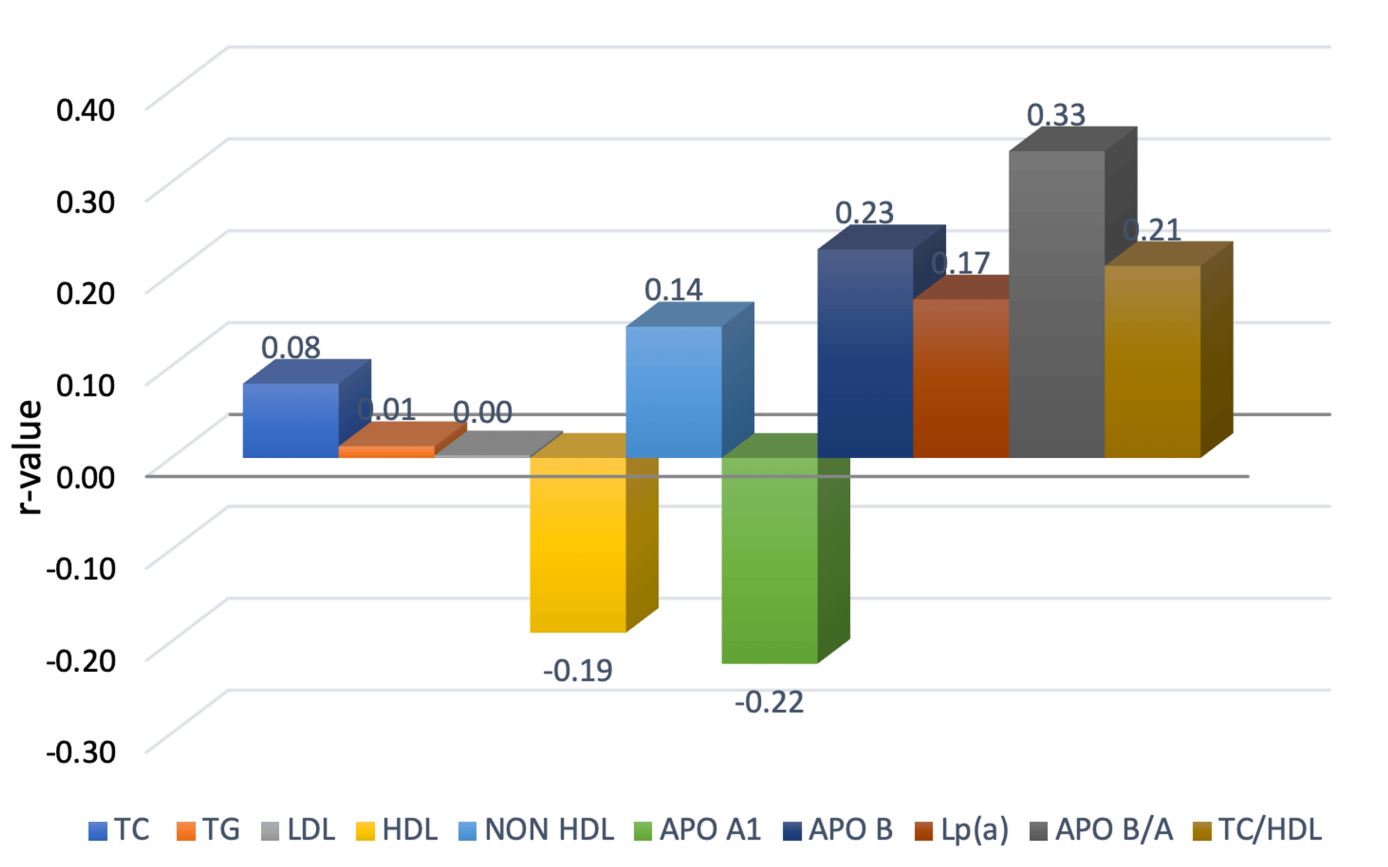
Correlation between lipid variables and SYNTAX score Apo A1: apolipoprotein A1; Apo B/A: apolipoprotein B/A; HDL: high-density lipoprotein; LDL: low-density lipoprotein; Lp(a): lipoprotein (a); TC: total cholesterol; TG: triglycerides

The majority of cases (n = 79, 79%) belonged to the low-risk class as per the SYNTAX score. Apo 1 (r-value = -0.22, p<0.05) correlated significantly in an inverse manner while Apo B (r-value = 0.23, p<0.05), Apo B/A (r-value = 0.33, p<0.01), and TC/HDL (r-value = 0.21, p<0.05) had a significant positive correlation with SYNTAX-I score class severity (Figure 3).

## Discussion

The majority of the patients in this study were male, which could be attributed to socioeconomic factors, as well as atypical presentation among females leading to underdiagnosing of the ACS burden in females. South Asians are more prone to develop CAD at relatively younger ages than their global counterparts, which was observed in this study, as well as in another study: the INTERHEART study by Yusuf et al. in 2004 [6]. Females presented at an older age than their male counterparts and it could be due to the protective effect of estrogen on their cardiovascular system. Smoking and hypertension were associated with CAD. This study also highlights the presence of hypertriglyceridemia, raised LDL, and low HDL in CAD. Misra et al. [7] showed that atherogenic dyslipidemia (comprising a combination of hypertriglyceridemia, low HDL, and raised LDL) is particularly prevalent in Indians.

Significant atherosclerosis was present in the majority of the cases, which emphasizes that atherosclerosis is the major etiology of CAD. SVD was the most common type of involvement, followed by DVD and TVD. The most common vessel involved was LAD, followed by RCA and LCX. Left main involvement was the least prevalent. LAD involvement is more common than RCA because of the hemodynamic differences in the left and the right coronary arteries. RCA flow is more uniform than the left main coronary artery during the cardiac cycle. Left coronary flow decreases during systole and increases significantly during diastole. This increase-decrease pattern in blood flow during the cardiac cycle produces oscillatory shear wall stress in the left coronary system. Also, anatomic configuration and the phasic motion between the right and the left arterial system modulate the local atherogenic environment, as observed in the study by Chatzizisis et al. [8].

The proximal segment was the most commonly involved, followed by the mid and distal segments. Ostial involvement was observed to be the least common involvement. Proximal involvement is seen most commonly because the proximal segments are more exposed to low shear stress than distal ones, as in the study by Rodriguez-Granillo et al. [9], due to high-velocity blood impacting against anatomical flow dividers producing flow turbulence as per Kimura et al. [10]. This low shear stress, in turn, causes loss of physiological flow-oriented alignment of the normal endothelial cells, increased expression of adhesion molecules, and cell junctions. McLenachan et al. showed that this all leads to increased permeability to macrophages and lipids [11].

Significant atherosclerosis was associated with TC, non-HDL, and Apo B/A levels. The role of TC in CAD is evident by the fact that cholesterol forms the core of atherosclerotic plaques, causing stenosis. Its association with CAD is also described by Kannel et al. and Bahulikar et al. [12,13]. Non-HDL represents the cholesterol content present in all the atherogenic lipoproteins, including LDL, and is considered a better predictor of CAD risk. Similar results were found in this study and it aligns with the findings of Bahulikar et al. and Di Angelantonio et al. [13,14]. Apo B100 is present in all atherogenic particles, while Apo A1 is part of the anti-atherosclerotic particles. Thus, the Apo B/A ratio represents the balance of atherogenic and anti-atherogenic particles in the blood. A higher ratio favors atherosclerosis, leading to CAD. Hence, a higher Apo B/A ratio has recently been seen as a better risk predictor of CAD, as in the studies by Karthikeyan et al., Kaneva et al., and McQueen et al. [15-17]. In this study, the Apo B/A ratio was significantly associated with the risk of CAD, and Apo B/A (p<0.01) was superior to any other lipid variable or TC/HDL ratio in terms of association with CAD risk. This finding is consistent with that of McQueen et al. [17] and Tamang et al. [18].

Apo B/A was significantly associated with LAD, LCX, and RCA involvement; however, it remains non-specific and, hence, in general, merely represents a better CAD risk predicament. TC and non-HDL demonstrate significant association with RCA involvement. McGill et al. [19] showed that LDL cholesterol concentration and non-HDL are positively associated with the extent of fatty streaks and raised lesions in the right coronary artery; however, their study involved a young population. Given continued lipid deposition and proliferation of smooth muscle and connective tissue, these fatty streaks and fibrous plaques increase in size and extent with time; some may undergo qualitative changes such as rupture, which exposes the blood to lipid-rich thrombogenic material and precipitates an occlusive thrombus, which in turn leads to ACS events. This might explain the significant presence of TC and non-HDL with RCA involvement, as found in this study.

A significant association of Apo B/A, TC, and non-HDL with mid and distal segment involvement was observed. The proximal segment is most commonly involved in CAD. Extension of the severity of CAD simply means the progression of atherosclerosis to diffuse and severe forms. This implies that atherosclerotic plaque that begins from the proximal segment will spread to nearby segments such as the mid and distal segments. TC, non-HDL, and Apo B/A are all associated with the extensiveness of CAD. This may explain why TC, non-HDL, and Apo B/A are all significantly associated with mid and distal segment involvement. Deng et al. [20] have reported that Apo B/A is associated with plaque rupture, erosion, and thrombus, a phenomenon that may cause the downstream segments to be involved. This might be another explanation for the significant Apo B/A association with mid-segment involvement.

Most of the previous studies have analyzed the correlation of lipid profile with the GENSINI or Friesinger score. Correlation with the SYNTAX score is relatively less studied. Apo A1 correlated inversely while Apo B, Apo B/A, and TC/HDL correlated positively with the SYNTAX score class. Apo A1 is part of all anti-atherogenic lipid particles and hence plays a protective role against atherosclerosis and is expected to be negatively correlated with the severity of CAD. On the other hand, Apo B is part of all atherogenic particles and would correlate positively with CAD severity. The finding of this study corroborates this logic. In this study, Apo B/A correlated significantly with the severity of CAD as assessed by the SYNTAX score. A higher ratio favors more cholesterol in plasma and serves as pro-atherosclerotic and, hence, a severe disease. Similar results were seen in previous studies by Yaseen et al. [21] and Hua et al. [22].

TC/HDL also favors severe atherosclerosis in the same vein as TC, while HDL favors anti-atherogenicity. Thus, the higher the ratio, the more severe the atherosclerosis. This is in line with a previous study by Lin et al. [23]. Between the two, Apo B/A correlated better than TC/HDL. This can be explained by the fact that Apo B represents atherogenic lipoprotein particles better than TC, and, similarly, Apo A represents all antiatherogenic lipoprotein particles better than HDL. Similar findings were also seen in previous studies by Tian et al. and Goswami et al. [24,25]. It is already well-known that the risk of CAD and its severity is based on lipid profile. This adds to this body of knowledge by exploring the association of lipids and coronaries in terms of the number of vessels involved, vessel involved, segment involved, and CAD severity by SYNTAX.

Limitations

This study, while providing valuable insights into the association between lipid parameters and SYNTAX score in ACS patients undergoing PCI, has several limitations. The relatively small sample size and single-center design may limit the generalizability of the findings to a broader population. Also, the cross-sectional nature of the study precludes the establishment of causal relationships and the assessment of long-term outcomes. Another significant limitation is the exclusion of patients who did not undergo angiography, potentially introducing a bias toward a more severe patient population. Future studies should aim to include a larger and more diverse cohort of ACS patients, especially those who did not undergo invasive procedures. Further research is needed to explore the potential of lipid-based stratification models to predict adverse outcomes in ACS patients. Prospective studies with longer follow-up periods can provide more robust evidence on the prognostic value of lipid parameters and their interaction with other clinical factors. By addressing these limitations and pursuing further research, a more comprehensive understanding of the role of lipids in ACS can be achieved, leading to improved risk stratification and personalized treatment strategies.

## Conclusions

This study explored the relationship between lipid profile and CAD severity in ACS patients. By analyzing angiographic parameters and SYNTAX scores, it identified a strong association between lipid parameters (TC, non-HDL cholesterol, Apo B/A ratio) and the extent of CAD, including vessel involvement, specific vessel involvement, and segmental disease distribution. The Apo B/A ratio showed a particularly robust correlation with CAD severity, suggesting its potential as a valuable prognostic marker. SYNTAX score was also influenced by lipid profile, with Apo B, Apo B/A, and TC/HDL positively correlating with disease severity. The study highlights the clinical value of comprehensive lipid assessments in managing ACS patients and suggests that targeting lipid abnormalities may lead to improved outcomes. However, the study's limitations necessitate further investigation to validate these findings in larger and more diverse populations. Future research should focus on prospective studies to establish causality between lipid profile and CAD progression and explore the potential of lipid-based risk stratification models for guiding treatment decisions.

## Figures and Tables

**Figure 4:**
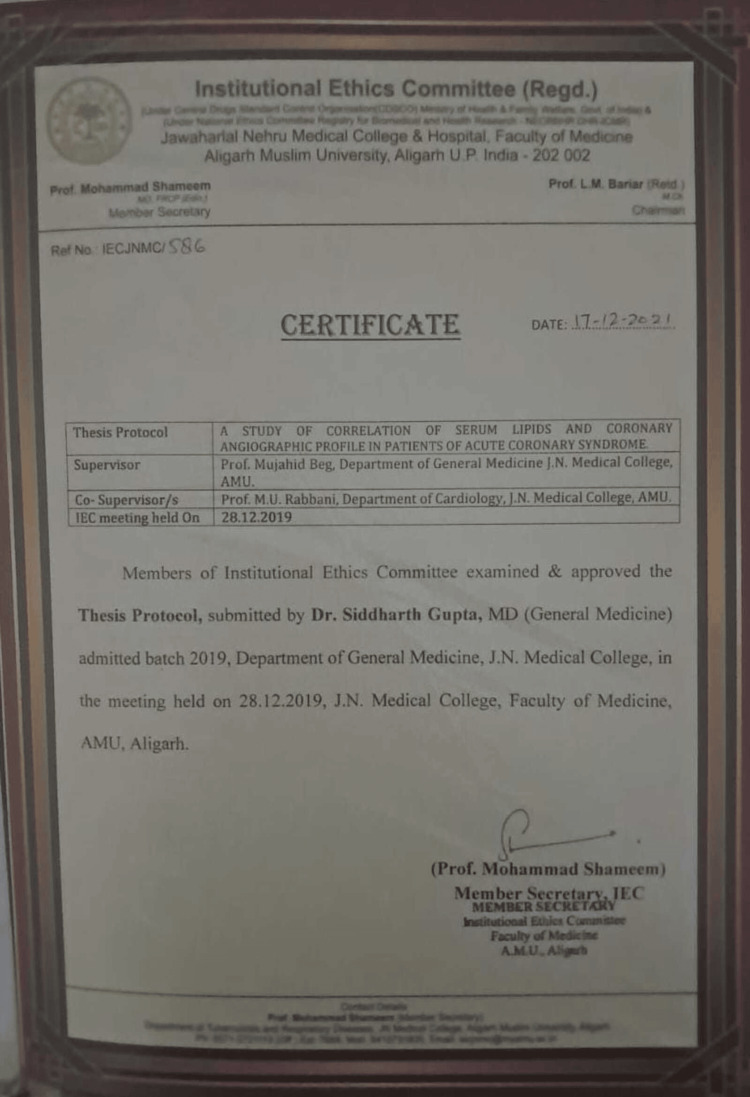
Institutional Review Board approval letter Note: The study was approved by the Institutional Review Board on 28.12.2019 as mentioned in the approval letter; however, because of the sad demise of one of the supervisors, another meeting was scheduled dated 17.12.2021 to replace the demised supervisor with the new one. The study data were not retrieved from the medical records

## Appendices

Relevant terms and their definitions

Dyslipidaemia: defined as the presence of any one of the following: TC >200 mg/dl, TG >150 mg/dl, LDL >100 mg/dl, non-HDL >130 mg/dl, HDL<40 in males and <50 in females (NCEP-ATP III guidelines), lipoprotein (a) >20 mg/dl (LAI guidelines), Apo B <80 (ESC guidelines).

Significant CAD disease was defined as stenosis involving ≥ 50% of the diameter of any epicardial vessel.

Severe lesions are termed as such if >70% of vessel lumen is involved in the left anterior descending artery (LAD), left circumflex artery (LCX), right coronary artery (RCA), and >50% in left main coronary artery (LMCA)

Based on the vessels involved, CAD is classified as:

Single vessel disease (SVD): ≥50% stenosis in one epicardial coronary artery with respect to reference vessel diameter.

Double vessel disease (DVD): ≥50% stenosis in two epicardial coronary arteries with respect to reference vessel diameter.

Triple vessel disease (TVD): ≥50 % stenosis in three epicardial coronary arteries with respect to reference vessel diameter.

The location of the lesion in the coronary vessel segment was noticed and is categorized as follows: ostial, proximal, mid, and distal.

1. RCA proximal: from the ostium to one-half the distance to the acute margin of the heart.

2. RCA mid: from the end of the first segment to the acute margin of the heart.

3. RCA distal: from the acute margin of the heart to the origin of the posterior descending artery.

4. LMCA: from the ostium of the left coronary artery to bifurcation into LAD and LCX.

5. LAD proximal: proximal to and including the first major septal branch.

6. LAD mid: LAD immediately distal to the origin of the first septal branch and extending to the point where LAD forms an angle (RAO view). If this angle is not identifiable, this segment ends at one-half the distance from the first septal to the apex of the heart.

7. LAD distal: terminal portion of LAD, beginning at the end of mid-segment and extending to or beyond the apex.

8. Proximal circumflex: main stem of circumflex from its origin of left main to and including the origin of the first obtuse marginal branch.

9. Mid circumflex: branch from trifurcating left main other than proximal LAD or LCX.

10. Distal circumflex: stem of circumflex distal to the origin of most distal obtuse marginal branch and running along the posterior left atrioventricular grooves.

Institutional Review Board approval letter
